# Presidential election results in 2018-2022 and its association with excess mortality during the 2020-2021 COVID-19 pandemic in Brazilian municipalities

**DOI:** 10.1590/0102-311XEN194723

**Published:** 2024-06-14

**Authors:** Everton Emanuel Campos de Lima, Lilia Carolina Carneiro da Costa, Rafael F. Souza, Cleiton O. da E. Rocha, Maria Yury Travassos Ichihara

**Affiliations:** 1 Universidade Estadual de Campinas, Campinas, Brasil.; 2 Instituto de Matemática, Universidade Federal da Bahia, Salvador, Brasil.; 3 Instituto Gonçalo Moniz, Fundação Oswaldo Cruz, Salvador, Brasil.

**Keywords:** Excess Mortality, COVID-19 Pandemic, Politics, Excesso de Mortalidade, Pandemia por COVID-19, Política, Exceso de Mortalidad, Pandemia de COVID-19, Política

## Abstract

We evaluated the hypothesis of an association between excess mortality and political partisanship in Brazil using municipal death certificates registered in the Brazilian Ministry of Health database and first-round electoral results of Presidential elections in 2018 and 2022. Considering the former Brazilian President’s stance of discrediting and neglecting the severity of the pandemic, we expect a possible relationship between excessive mortality rates during the COVID-19 health crisis and the number of municipal votes for Bolsonaro. Our results showed that, in both elections, the first-round percentage of municipal votes for Bolsonaro was positively associated with the peaks of excess deaths across Brazilian municipalities in 2020 and 2021. Despite the excess mortality during the pandemic, the political loyalty to Bolsonaro remained the same during the electoral period of 2022. A possible explanation for this is linked to the Brazilian political scenario, which presents an environment of tribal politics and affective polarization.

## Resumo

Usando dados municipais em declarações de óbito registrados no Ministério da Saúde e resultados eleitorais do primeiro turno das eleições presidenciais de 2018 e 2022, avaliamos a hipótese de que há associação entre excesso de mortalidade e partidarismo político no Brasil. Dada a postura do ex-presidente brasileiro de desacreditar e negligenciar a gravidade da pandemia, esperamos que haja possivelmente uma relação entre as taxas excessivas de mortalidade durante a crise sanitária da COVID-19 e o número de votos municipais para Bolsonaro. Nossos resultados mostraram que, em ambas as eleições, o percentual de votos municipais no primeiro turno para Bolsonaro foi positivamente associado aos picos de excesso de mortes nos municípios brasileiros em 2020 e 2021. Mesmo com o excesso de mortalidade durante a pandemia, a lealdade política de Bolsonaro não diminuiu durante o segundo período eleitoral em 2022. Uma possível explicação para isso está ligada ao cenário político brasileiro, que vive um ambiente de política tribal e polarização afetiva.

## Resumen

A partir de datos municipales sobre certificados de defunción registrados en el Ministerio de Salud de Brasil y de los resultados electorales de la primera vuelta de las elecciones presidenciales de 2018 y 2022, se evaluó si existe una asociación entre el exceso de mortalidad y el partidismo político en Brasil. Ante la postura del ex presidente brasileño de desacreditar y desatender la gravedad de la pandemia, probablemente exista una relación entre las altas tasas de mortalidad durante la crisis de salud del COVID-19 y el número de votos municipales para Bolsonaro. Los resultados demostraron que, en ambas elecciones, el porcentaje de votos municipales en la primera vuelta para Bolsonaro estuvo asociado positivamente con los picos de alta de muertes en los municipios brasileños para el período 2020-2021. Incluso con la alta mortalidad durante la pandemia, la lealtad política de Bolsonaro no disminuyó durante el segundo período electoral en 2022. Una de las posibles explicaciones es que esto se vincula al escenario político brasileño, que vive una política tribal y polarización afectiva.

## Introduction

In the first months of 2020, COVID-19 infections emerged worldwide. As the disease emerged from China, it quickly spread to Europe and the United States ^1^ ^,^ ^2^ ^,^ ^3^. Consequently, many health services were disrupted, and families experienced many changes in their daily lives due to the pandemic. At the beginning of the outbreak, the scientific community’s knowledge about the COVID-19 virus and how it spread was scarce ^4^. Despite incipient knowledge, most nations introduced interventions to reduce the spread of the virus as a direct response to this global health crisis.

At the same time, during the COVID-19 pandemic, the worldwide political scenario presented populist governments and polarized societies, and many political opinions and policy issues were subjected to this divided tribal politics (a term that holds a general negative connotation in a political context, which can also refer to discriminatory behaviors or attitudes towards out-groups based on in-group loyalty), sometimes with tragic consequences to the overall health of society ^5^. In the U.S., for instance, during the pandemic, the former President Donald Trump repeatedly tried to silence and discredit science advice in general ^6^. Wallace et al. ^7^ pointed that Trump’s actions brought terrible consequences for mortality levels in the U.S., and found a substantially higher excess death rate for Republicans when compared to Democratic voters.

Stuenkel ^8^ argues that the polarized Brazilian society started in 2013, when the country experienced massive demonstrations in major cities. Initially motivated by economic issues, the protests soon questioned inadequate social services and corruption involving the ruling Workers’ Party (PT). In 2018, Jair Bolsonaro, candidate of the Social Liberal Party (PSL) and a long-time right-wing congressman, positioned himself against PT and as an anti-establishment candidate, beating the PT’s presidential candidate in the runoff 2018 Brazilian election ^8^.

Baptized as Tropical Trump in 2018 ^9^, the official government responses to the pandemic were similar to the U.S., presenting heterogeneity and lack of coordination ^4^. The populist leader Jair Bolsonaro and his political alignment view and rhetoric found a place with other populist leaders worldwide, particularly President Trump ^4^ ^,^ ^10^. The former President Bolsonaro disregarded the pandemic on many occasions, explicitly challenging the scientific community’s and the World Health Organization’s (WHO) recommendations ^4^. Due to his controversial speeches, pleading for relaxing social-distancing measures, and encouraging people to disrespect lockdown measures ^4^, the Brazilian former President attracted international media attention. At the same time, the numbers of infections and deaths in Brazil became higher in many moments of the pandemic. With the U.S., Brazil became one of the leading countries in the infection rates of COVID-19 ^11^. Ajzenman et al. ^4^ showed that Bolsonaro’s words and actions caused a substantial effect on relaxing social distancing in the country’s municipalities, which might have increased the spread of infection. During the pandemic, Bolsonaro showed a consistent pattern of defiance against scientific advice and interventions that could prevent the spread of COVID-19 ^12^ ^,^ ^13^.

Moreover, other studies have recently shown significant relations between political ideology and different aspects of COVID-19. For example, Xavier et al. ^14^ investigated the association between COVID-19 deaths and political partisanship in Brazil. They found that municipalities with more support for Jair Bolsonaro during the 2018 elections presented higher COVID-19 mortality rates, even controlling for health and socioeconomic conditions. Furthermore, Hastenreiter Filho & Cavalcante ^15^ identified correlations between COVID-19 mortality in Brazilian municipalities and socioeconomic and political variables using cross-sectional data. The results point to positive and significant associations between mortality per million inhabitants and factors such as gross domestic product (GDP) per capita, municipalities pertaining to a metropolitan area, percentage of older adults, urbanization, and percentage of votes for Jair Bolsonaro in the 2018 elections. The regional analysis reinforces these conclusions, despite some regions showing variations in p-values. In summary, these studies contribute to understanding the factors linked to COVID-19 mortality in Brazilian municipalities, highlighting the importance of the percentage of votes for Jair Bolsonaro in the 2018 elections as a relevant variable to be considered in analyses and policies to combat the pandemic.

Moreover, vaccine hesitancy impacts significantly global public health, and political views can influence vaccine acceptance rates. For instance, Seara-Morais et al. ^16^ used an ecologic study design in Brazil to verify the association between political ideology and COVID-19 vaccine. They found that the percentage of votes for the right-wing candidate Jair Bolsonaro in the presidential elections of 2018 and 2022 was significantly and inversely associated with COVID-19 vaccine uptake, considering the adjustment for the sociodemographic characteristics of the voters ^16^. Corroborating this analysis, Backhaus et al. ^17^ explored the association between the voting far-right and COVID-19 vaccine hesitancy in 21 European countries. They found that far-right voters were 2.7 times more likely to be COVID-19-vaccine hesitant than center voters ^17^. This result is similar among European countries and persisted even after controlling for institutional trust and social participation.

Other studies analyzed the relationship between psychological aspects, COVID-19, and political ideology. Wallace et al. ^18^ verified the link between political ideology (conservatism and liberalism) and perceptions of personal responsibility and blame associated with COVID-19. They stated that “*conservative ideology was consistently linked to individual blame and responsibility, with those endorsing conservative ideology agreeing less with explanations of racial disparities based on structural inequalities and agreeing more with explanations for racial disparities based on personal blame and responsibility*” ^18^ (p. 1). Panish et al. ^19^ studied the relationship between openness, one of the big five personalities, and COVID-related behaviors, mediated by liberal (vs. conservative) ideological self-placement. According to these findings, openness does not always directly cause pandemic-related attitudes and actions, as some earlier research has indicated. Conversely, since they are more likely to receive and accept appeals from information sources connected to the political left, highly-open people may be more likely to adopt attitudes and engage in actions to limit the COVID-19 spread ^19^. Thus, these outcomes highlight the role information settings play in creating connections between attitudes and political psychology.

Another important aspect of the relationship between COVID-19 and political ideology is the misperceptions or levels of media literacy about a pandemic ^20^. Positive correlations were found between COVID-19 misperceptions and conservative ideology, younger age, consumption of conservative media, information gleaned from social media, and information gleaned from Donald Trump ^20^. In the meantime, misperceptions about COVID-19 were adversely correlated with facts gathered from local media and scientists. Moreover, conservative stances presented different misperceptions about COVID-19 from liberals, who were more media-literate overall. These findings showed that, despite self-reported media literacy, conservatives seem to believe in an echo chamber of false information, hence, it is necessary to provide greater exposure to opposing views ^20^.

In sum, the political tensions and poor conditions in Brazil have raised concerns about the impact of the pandemic on the number of deaths. This led to several studies on life expectancy reductions and excess mortality in the country ^11^ ^,^ ^12^ ^,^ ^21^. In this context, we extend this discussion and try to understand the associations of the electoral results in Brazilian municipalities during the 2018 and 2022 elections, with the excess mortality peaks verified at the municipal level during the 2020-2021 pandemic periods.

## Data and methods

Monthly death toll data were obtained from the Brazilian Ministry of Health, launched in the Brazilian Mortality Information System (SIM) ^22^. The SIM is a registration system developed by the Brazilian Health Informatics Department (DATASUS). This system was implemented in 1979, and the data are collected routinely immediately after each death in a standardized document (death certificates). This database contains many essential variables for the mortality study, such as socioeconomic, sociodemographic, and health information.

Other socioeconomic variables were provided by other Brazilian Government entities, including the Brazilian Ministry of Social Security, Brazilian Ministry of Citizenship, Institute of Geography and Statistics, and Brazilian Ministry of Labor and Employment. Data on electoral results were obtained from the Brazilian Superior Electoral Court. Moreover, religious affiliations were controlled (as percentage of Pentecostals living in the municipality). Evangelical groups, especially Pentecostals, found themselves in the liberal-conservative discourse elements that attracted their electoral support. This religious group is commonly associated with Bolsonaro supporters ^23^. Box 1 summarizes the variables and their sources.

**Box 1:** Variables used to analyze excess mortality in Brazilian municipalities during the COVID-19 pandemic, 2020-2021.

PARAMETER	DESCRIPTION	SOURCE
Response variable		
Excess mortality during 2020 and 2021	Pscore, or the percentage increase in the monthly number of deaths between April 2020 and June 2021 in comparison with five previous years	DATASUS ^38^
Variable of interest		
First-round votes for Bolsonaro in 2018 and 2022 and first-round votes for Worker’s Party (PT) in the same years	% votes to specific candidate divided by total votes (white and null included in the total)	Ipeadata ^39^ ^,^ ^40^
Demographic control variable		
% age retirement in 2021	The number of retirees at 65 (for males) and 62 (for females) years old by the total of retirees.	Brazilian Ministry of Social Security ^41^
Socioeconomic control variables		
GDP in 2019	GDP at current prices	IBGE ^42^
Extreme poverty ratio in 2020 and 2021	The ratio of person/family in extreme poverty situation during the mortality peaks in 2020 and 2021	Secretariat for Evaluation, Information, Management and Simple Registry ^43^ ^,^ ^44^
Unemployment numbers	Total number of unemployed people in four months previous to the peak of excess mortality	Brazilian Ministry of Labor and Employment ^45^
Material deprivation index	An index measuring household material deprivation in 2010	IBGE ^46^
Health service control variables		
Health facilities	The number of health establishments registered during the pandemic peaks	DATASUS ^47^ ^,^ ^48^
Health outpatient clinics	The number of health outpatient clinics during the pandemic peaks	DATASUS ^49^ ^,^ ^50^
Hospitalization	The number of hospitalizations approved during the pandemic peaks	DATASUS ^51^ ^,^ ^52^
ICU beds	Number of intensive care unit beds during the peaks of the pandemic	DATASUS ^53^ ^,^ ^54^
Vaccination coverage	Percentage of vaccine coverage in the municipalities in 2020 and 2021	DATASUS ^55^
Cultural variables		
% Pentecostals	Percentage affiliation of Pentecostals across municipalities in 2010	IBGE ^46^

DATASUS: Brazilian Health Informatics Department; GDP: gross domestic product; IBGE: Brazilian Institute of Geography and Statistics; ICU: intensive care units.

The selected variable of interest is the electoral outcomes in the first-round elections for Bolsonaro (PSL candidate in 2018 and PL - Liberal Party - candidate in 2022) and their biggest opposition party, the PT. First-round political results are aimed since they better capture the ideological groups attached to each figure or political party.

Not all control variables were used in the next section of regression models. Variables were selected based on the correlation matrices and variance inflation factor (VIF) criteria to identify potential multicollinearities in the models. The VIF statistic was employed with a cutoff value set at 10, as recommended by James et al. ^24^. In essence, variables with VIF values exceeding 10 were systematically excluded from the model. In the conclusive model, all variables demonstrated values below five. Notably, a VIF below five indicates a low correlation between predictors ^24^.

As a final selection, our complete models account for the following municipal variables: First-round votes to PSL and PL (Bolsonaro’s party in 2018 and 2022 elections, respectively) and PT; GDP; extreme poverty ratio; material deprivation index; % retirees by age; % Pentecostals; number of health facilities; number of health outpatient clinics; and longitude and latitude coordinates.

### Estimation of excess mortality

The definition of excess mortality follows the definition by Mathieu et al. ^25^ and Muellbauer & Aron ^26^. The excess mortality is given as P-scores, representing average monthly death data from 2015 to 2019 compared with the number of deaths in the pandemic months of 2020 and 2021 for each municipality. In equation terms, the P-score is defined as:

$$Pscore=\left[\frac{\left({Deaths}_{Period\;2020-2021}-{Average\;Deaths}_{Period2015-2019}\right)}{{Average\;Deaths}_{Period2015-2019}}\right]\times 100$$

The P-score is a common measure used to express the excess of mortality, but it holds limitations. The first limitation is related to the measure (5-year average death); it disregards trends in population size or mortality and represents a crude measure of expected mortality ^25^ ^,^ ^26^ ^,^ ^27^. Despite this limitation, when analyzing the development of deaths in the past five years in all sub-national areas of Brazil, mortality did not show an expressive trend (decrease or increase in mortality) over time. A second limitation is related to death reports and their registers. Delays in reporting deaths make mortality data provisional and incomplete after a death occurs. However, this issue is less problematic since, according to Queiroz et al. ^28^, the completeness of death count coverage improved over time throughout Brazil, especially in lesser-developed regions. This happened probably due to public investment in health data collection. However, later death registrations could still affect the estimates of excess mortality downwards. This could occur if it is assumed that the COVID-19 crisis may have hindered registration systems. To reduce the problem of later registration of events, this study employed the same strategy that Lima et al. ^11^ applied and considered the most up-to-date death information. Therefore, the latest available data on mortality was used, as published by the Brazilian Ministry of Health. Specifically, data on death published in 2022 were collected for the study estimates.

### Spatial dependence models

Spatial regression models were employed to understand underlying factors associated with excess mortality across Brazilian municipalities. These models considered that the assumption of independent observations, present in linear regression models, in the context of area data, is simplified and unappropriated due to the possibility of spatial dependence between the error terms ^29^ ^,^ ^30^. Spatial dependence are found when values observed in one geographic unit depend on the significance of neighboring observations in nearby areas ^30^. Notably, various specifications exist for spatial dependence modeling, including the spatial moving average error model, spatial cross-regressive model, spatial cross-regression model with spatial moving average error, spatial Durbin model, spatial lag model with spatial moving average error, and spatial Durbin error model, among others. In this study, however, the most widely used and popular version of spatial autoregressive models were employed, namely: spatial simultaneous autoregressive models ^31^.

In total, this study presents two main specifications regarding spatial dependence: (i) spatial lag models and (ii) spatial error models. The spatial lag indicates a spatial correlation (dependence) in the response variable. This is related to neighborhood effects or spatial externalities that cross the borders of the geographic units and appear in the response variable ^29^ ^,^ ^30^.

The equation, as an extension of linear regressions, of the spatial simultaneous autoregressive models allow observations of the dependent variable y in area *i* (*i* = 1,..., *n*) to depend on observations in neighboring areas *j* ≠ *i* ^30^, as shown in:

$$Y_{i}=\rho \;\sum_{j=1}^{n}W_{ij}Y_{j}+\sum_{q=1}^{Q}X_{iq}\beta_{q}+\varepsilon_{i}$$

In which *W*_*ij*_ is the (*i*,*j*)th element of the n-by-n spatial weights matrix *W*. The *X*_*iq*_ is an observation on an explanatory variable, with *q* = 1,...,*Q* (including a constant, or one), *β*_*q*_ represents a vector of several regression coefficients, and *ε*_*i*_ the error term ^30^. The scalar *ρ* is a parameter that determines the strength of the spatial autoregressive relation between *y*_*i*_ and *Σ*_*j*_ *W*_*ij*_ *y*_*j*_ , a linear combination of spatially related observations based on nonzero elements in the *i*th row of *W* ^30^.

The spatial error models are considered when spatial dependence is identified as the error term or the errors from different areas may display spatial covariance. In theoretical terms, spatial error dependence may arise, for example, from unobservable latent variables that are spatially correlated, or in the cases where area boundaries do not accurately reflect neighborhoods ^22^. The mathematical equation is given by:

$$\varepsilon_{i}=\;\lambda \sum_{j=1}^{n}W_{ij}\varepsilon_{j}+U_{i}$$

In which *λ* is the autoregressive parameter, and *u*_*i*_ is a random error term. To identify the appropriate model, the Lagrange multiplier test diagnostics for spatial dependence (Table 1) was applied. In the following sections, the empirical results are presented, first analyzing the correlation between votes for Bolsonaro and municipal excess mortality.

**Table 1:** The Lagrange multiplier test diagnostics for spatial dependence and regression models to explain municipal excess mortality in Brazil during the 2020-2021 COVID-19 pandemic associated with Bolsonaro’s first-round votes in 2018-2022.

Lagrange multiplier test diagnostics for spatial dependence, Bolsonaro’s first-round votes 2018-2022								
Type of spatial dependency test	2018 votes and 1^st^ peak mortality		2022 votes and 1^st^ peak mortality		2018 votes and 2^nd^ peak mortality		2022 votes and 2^nd^ peak mortality	
	Statistic	p-value	Statistic	p-value	Statistic	p-value	Statistic	p-value
Lagrange multiplier error model	21.28	0.000	23.35	0.000	25.27	0.000	25.92	0.000
Lagrange multiplier lag model	18.82	0.000	20.22	0.000	18.92	0.000	19.52	0.000
Robust Lagrange multiplier error model	2.88	0.090	3.91	0.048	7.24	0.007	7.26	0.007
Robust Lagrange multiplier lag model	0.41	0.522	0.79	0.375	0.89	0.344	0.86	0.354
Regression models to explain municipal excess mortality in Brazil in the function of first-round votes for Bolsonaro								
Control variables	2018 PSL votes - 1^st^ peak excess deaths				2022 PL votes - 1^st^ peak excess deaths			
	Linear model		Spatial error model		Linear model		Spatial error model	
	β	p-value	β	p-value	β	p-value	β	p-value
Intercept	36.83	0.002	36.73	0.003	41.11	0.001	41.17	0.001
% first-round votes	0.62	0.000	0.61	0.000	0.51	0.000	0.52	0.000
Material deprivation index	2.97	0.263	3.15	0.258	0.95	0.704	1.35	0.609
GDP	0.09	0.573	0.08	0.640	0.09	0.570	0.08	0.640
Extreme poverty ratio	-4.16	0.223	-3.78	0.281	-3.80	0.265	-3.47	0.323
% age retirement	0.08	0.354	0.07	0.442	0.05	0.558	0.04	0.668
% Pentecostals affiliated	0.55	0.001	0.57	0.002	0.56	0.001	0.58	0.001
Health establishments	-0.004	0.405	-0.003	0.473	-0.004	0.401	-0.003	0.474
Health outpatient clinics	0.01	0.663	0.01	0.666	0.01	0.621	0.01	0.631
Longitude	0.20	0.351	0.21	0.353	0.22	0.311	0.25	0.295
Latitude	1.26	0.000	1.23	0.000	1.24	0.000	1.21	0.000
	R^2^ = 0.019		Lambda: 0.017 *		R^2^ = 0.018		Lambda: 0.018 *	
	AIC: 63,172		AIC: 63,154		AIC: 63,200		AIC: 63,180	
Control variables	2018 PSL votes - 2^nd^ peak excess deaths				2022 PL votes - 2^nd^ peak excess deaths			
	Linear model		Spatial error model		Linear model		Spatial error model	
	β	p-value	β	p-value	β	p-value	β	p-value
Intercept	-9.85	0.587	-10.87	0.573	-6.07	0.740	-6.98	0.719
% first-round votes	0.64	0.001	0.64	0.001	0.48	0.006	0.49	0.009
Material deprivation index	-9.36	0.022	-8.52	0.047	-11.98	0.002	-10.98	0.007
GDP	0.20	0.436	0.22	0.404	0.20	0.436	0.22	0.405
Extreme poverty ratio	9.27	0.081	8.40	0.124	9.96	0.060	9.00	0.099
% age retirement	0.02	0.886	0.02	0.889	-0.01	0.929	-0.01	0.931
% Pentecostals affiliated	1.02	0.000	0.95	0.001	1.05	0.000	0.98	0.000
Health facilities	-0.004	0.544	-0.005	0.470	-0.004	0.539	-0.005	0.468
Health outpatient clinics	-0.01	0.679	-0.01	0.721	-0.01	0.708	-0.01	0.745
Longitude	-0.70	0.032	-0.79	0.027	-0.70	0.035	-0.79	0.031
Latitude	0.63	0.060	0.58	0.104	0.60	0.072	0.55	0.123
	R^2^ = 0.037		Lambda: 0.019 *		R^2^ = 0.036		Lambda: 0.019 *	
	AIC: 67,950		AIC: 67,927		AIC: 67,979		AIC: 67,956	

AIC: Akaike information criterion; GDP: gross domestic product; PL: Liberal Party; PSL: Social Liberal Party.

Sources: Brazilian Health Informatics Department (DATASUS) ^38^, Ipeadata ^39^ ^,^ ^40^, Brazilian Ministry of Social Security ^41^, Secretariat for Evaluation, Information, Management and Simple Registry ^43^ ^,^ ^44^ and Brazilian Institute of Geography and Statistics (IBGE) ^42^ ^,^ ^46^.

Note: models were also estimated for the largest opposition party (Workers’ Party - PT), although the results are presented as Supplementary Material (Material Suplementar(https://cadernos.ensp.fiocruz.br/static//arquivo/supl-e00194723_1445.pdf). Beta coefficients for PT vote, in this case, exhibited a negative relationship with excess mortality during the same periods.

* p-value < 0.001.

## Results

### Descriptive analyses

As a first look, we compared the spatial correlation between electoral results in 2018 and 2022. We found that the correlation between municipality results is almost perfect (0.98) when we compared electoral outcomes for Bolsonaro between 2018 and 2022 (Supplementary Material. Figure S1, panel A. Material Suplementar(https://cadernos.ensp.fiocruz.br/static//arquivo/supl-e00194723_1445.pdf). 

The PT showed a strong correlation between its votes in the two elections (0.92) (Supplementary Material. Figure S1, panel B. Material Suplementar(https://cadernos.ensp.fiocruz.br/static//arquivo/supl-e00194723_1445.pdf). Thus, this strong positive association between votes in 2018 and 2022 indicates that the spatial electoral distribution in Brazil is almost unchanged during these four years. Another compelling finding is the polarized electoral results that place PT and Bolsonaro in distinguished points of the Brazilian political spectrum. Moreover, this political polarization has increased between the two elections of 2018 and 2022 (the correlation coefficient was -0.91 in 2018 and became -0.99 in 2022).

**Figure 1:**
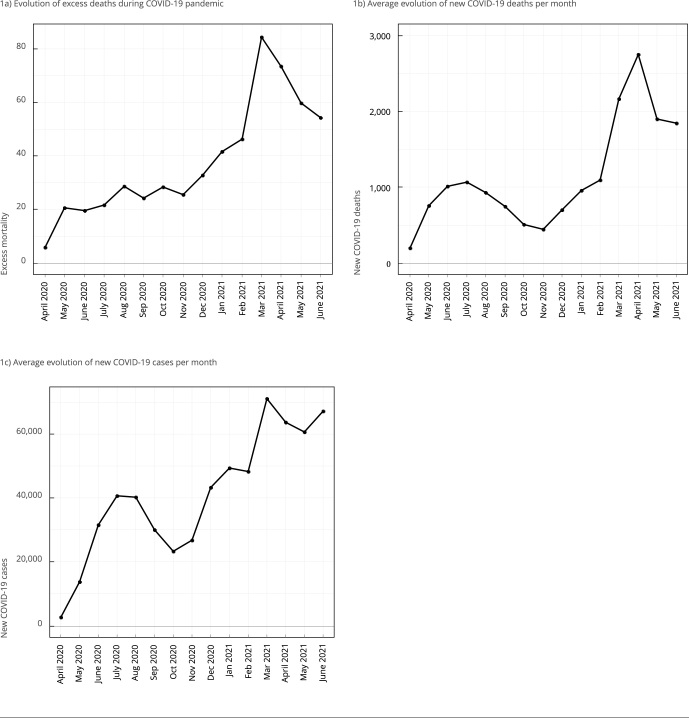
Pattern of temporal evolution of COVID-19 cases, deaths, and excess mortality in 2020 and 2021, Brazil.

Source: Our World in Data ^33^ and Brazilian Health Informatics Department (DATASUS) ^38^.

The next question to answer is whether there is a correlation between the first-round elections results and the municipal excess mortality rate during the COVID-19 pandemic in Brazil. Firstly, we analyzed the moments when mortality peaked. According to the Brazilian National Council of Health Secretaries ^32^, in 2020 and 2021, the country experienced 23% and 44% excess mortality, respectively, and COVID-19 accounted for approximately 13% and 23% of the deaths during these years ^22^ ^,^ ^33^. Therefore, we may assume a temporal correlation between excess mortality, infection cases, and deaths by COVID-19 (Figure 1).

Figure 1 shows that cases and deaths by COVID-19 peaked from July to August 2020 (first peak) and from March to April 2021 (second peak). The excess mortality followed a similar temporal pattern, peaking from July to August 2020 and from March to April 2021. In further analysis, we chose these date points, corresponding to excess mortality first and second peaks during the COVID-19 pandemic.

**Figure 2:**
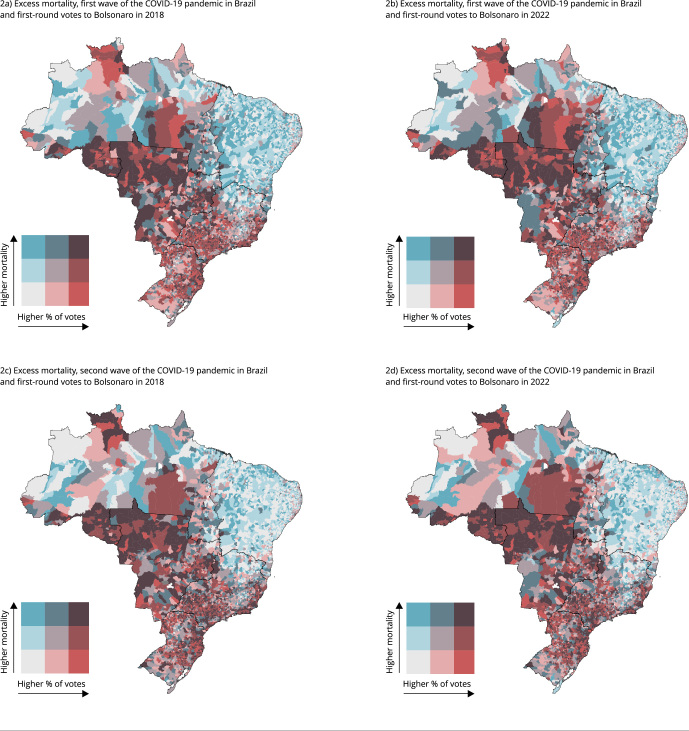
Maps of excess mortality across Brazilian municipalities, first and second waves of the COVID-19 pandemic, and first-round votes for Bolsonaro in 2018 and 2022.

Source: Brazilian Health Informatics Department (DATASUS) ^38^ and Ipeadata ^39^ ^,^ ^40^.

Figure 2 presents the excess mortality peaks (in August 2020 and April 2021) and Bolsonaro voters in bivariate maps. With this, we aimed to find bivariate spatial correlations between the excess mortality within these pandemic peak dates and the percentage of votes for Bolsonaro simultaneously. This time, we centered our analysis only on Bolsonaro voters, but in further spatial modeling, we also introduced the electoral results of PT.

In August 2020 (first wave), we observed a positive association between municipal votes for Bolsonaro and the excess mortality. In the areas of Central-West Brazil and parts of the Northern Amazon, we found many hotspots of excess mortality associated with a higher percentage of votes for Bolsonaro. This is verifiable in both years of 2018 and 2022. This last finding is expected given that the spatial correspondence between Bolsonaro’s votes in these two elections is highly positively correlated. However, this relationship between votes and excess mortality is still unclear. At the same time, in many municipalities in the South and Southeast of Brazil, where Bolsonaro had many voters, we found no excess mortality in August 2020. Moreover, in the Northeast, although we found few or no excess mortality in many places, we also identified many municipalities with excess mortality and few votes for Bolsonaro between the two elections.

The spatial relationship between voting and excess mortality becomes clear when we expand the analysis to the second peak of the pandemic (Figures 2c and 2d). In Northeastern Brazil and some municipalities in Northern Brazil, we hardly observe excess mortality. Moreover, in these places, Bolsonaro had less electorate percentage in the first round of elections in 2018 and 2022.

During that time, the Brazilian Central-West continued to present high percentage of Bolsonaro’s electorate. Meanwhile, in many municipalities in the South and Southeast, areas with many presidential voters, the excess of mortality has grown compared to the first wave of the pandemic. From these empirical findings, we can partially conclude that the spatial distribution of excess deaths was partially related to the percentage of municipal votes for Bolsonaro. However, part of these encountered relationships may be biased and must be controlled by other municipal confounding factors such as economic and health information.

### Spatial models results

Table 1 shows the regression models estimated to explain excess mortality in Brazil during the two pandemic waves, August 2020 and April 2021. We estimated both spatial and linear regression models, with the latter interpreted in cases without spatial dependence. Regarding the model selection, we employed the Lagrange multiplier test diagnostics for spatial dependence based on the following criteria: initially, we examined the Lagrange multiplier test for both lag and error terms; if both Lagrange multiplier test statistics were found to be significant, we subsequently considered the robust forms of the test statistics ^34^. Typically, only one is significant; in such cases, we estimated the regression model corresponding to the identified spatial dependence ^34^. In instances where no robust Lagrange multiplier test was found to be statistically significant, our analysis shifted to the linear regression model interpretations.

In Table 1, the Lagrange multiplier test diagnostics reveal spatial autocorrelation in the error term in almost all estimated models, indicating that the most suitable model incorporates a spatial error term. Furthermore, we also observed that linear and spatial error models did not differ significantly from each other.

In all models, after controlling for economic and health confounding factors, municipal votes for Bolsonaro were positively associated with excess deaths in that area. Considering the 2018 election results, we observed a strong association between excess deaths at the two peaks of the pandemic. In addition, the coefficients of association between Bolsonaro’s municipal voters and excess mortality presented no significant difference between the two pandemic peaks. This positive correlation between votes and excess deaths was expected to have a downward impact, and harm Bolsonaro’s political performance in the subsequent presidential election. However, in 2022, the electoral results continued to be positively associated with the excess of deaths in the municipalities. This time, the coefficients lost some of their explanatory power compared to the four previous years of election, but it continued to be highly correlated with excess mortality.

In general terms, each 1% increase in municipal votes for Bolsonaro from 2018 to 2022 corresponded to a rise of 0.48% to 0.64% in municipal excess mortality during the COVID-19 pandemic peaks. Another interesting fact is that none of the control variables showed a constant significant relationship with excess deaths. The only exception was the percentage of Pentecostals in the municipalities that were also positively related to the excess of deaths during pandemic peaks.

Moreover, we designed a contrafactual approach and introduced municipal votes that were seen as against Bolsonaro. For this, we selected the municipal first-round electoral outcomes of Bolsonaro’s most prominent opposition party, the PT (Supplementary Material. Table S1. Material Suplementar(https://cadernos.ensp.fiocruz.br/static//arquivo/supl-e00194723_1445.pdf). The spatial models show that votes against Bolsonaro were negatively associated with excess mortality. However, this was only visible at the second peak (correlating with votes in 2018) and during the two excess mortality peaks and vote outcomes of 2022.

## Discussion and conclusion

In 2018, Jair Bolsonaro won the Brazilian presidential elections and defeated, in the second round, his opponent from the PT, breaking a cycle of four elections won by the PT. Similarly to Donald Trump, the former American President, two years before the elections, Bolsonaro presented himself as a political outsider and an impetuous nationalist leader ^9^. Both political persons showed similarities, including the overlap in their populist rhetoric, authoritarian view of executive leadership, and use of social media to micro-target voters ^9^ ^,^ ^35^.

From March 2020, the country faced one of its most significant health crises, the SARS-CoV-2 pandemic. As a result, Brazil experienced many deaths and infections from this new virus. Similarly to other authoritarian world leaders, Bolsonaro adopted a stance of disbelief and discredited the possible mortality impacts that COVID-19 could bring to Brazilian society. Following the U.S. President Donald Trump’s footsteps, the political debate centered its discussions on the dichotomy of the economy versus the adoption of severe health measures. Although some studies show that social distancing measures did not compromise the economy of municipalities ^36^, the former Government’s disbelief delegated the fight against the pandemic to the Brazilian Federative Units and municipal spheres of the country ^4^.

As a result, Brazil became, at certain moments, one of the world’s epicenters in terms of mortality and infections from COVID-19. Following the incipient literature on the subject, we sought to understand whether there was a relationship between excess mortality during the peaks of the pandemic and Brazilian electoral preferences. Our analysis showed that, in general, municipalities with a high percentage of Bolsonaro voters were positively correlated with excess mortality during the COVID-19 pandemic. Thus, we found that the electoral spatial distribution of Bolsonaro voters was related to more excess mortality during the peaks of the pandemic. One explanation for this correlation may be linked to the role played by Bolsonaro as a public figure and influencer of his electorate. The disbelief about the harmful effects of the pandemic, the nonacceptance of wearing face masks, the initial resistance to purchase vaccines, and the slow implementation of an immunization campaign could be some of the reasons for this association between Bolsonaro’s votes and excess mortality. In other words, the vote can represent a set of ’attitudes of the electorate in line with the actions of its political leader. Still, it may also reflect the inadequate health measures adopted by local governments (municipalities) where Bolsonaro had a large number of votes.

The large number of deaths were expected to result in electoral loss in a subsequent election for Bolsonaro. However, this was not verified in 2022, when we continued to observe a strong and positive association between municipal voting for Bolsonaro and excess deaths. In the same way, the electorate against Bolsonaro, captured in PT votes, was negatively related to the excess mortality among Brazilian municipalities. Votes for the PT are expected to represent a less conservative population, which apparently adhered more to preventive measures, or a population that trusted more the health institutions. On the other hand, a second, perhaps more plausible explanation to both phenomena (maintenance of Bolsonaro electorate between elections and Bolsonaro’s opposition) would be linked to the concepts of “tribal politics” and “affective polarization” ^5^ ^,^ ^37^.

Tribal politics represent a political environment dominated by voters whose overriding concern is “those with us and those against us” and who support candidates representing their ethnic, religious, or ingroup beliefs regardless of the policy they promote ^5^. As pointed out by Dreyfuss et al. ^5^ the resurgence of tribal politics in recent years appears to shape a wide range of policies, from immigration and international trade to income redistribution, the rule of law, and even the responses to a pandemic and climate change. In total, three conditions are necessary to emerge from tribal politics: the first, from economic order, is related to socioeconomic and income distribution. The second relates to cultural elements that accentuate rivalries or increase the pride conferred by ethnic affiliation, which tends to expand the tribal vote base. The final condition is related to institutional and demographic factors and the share of each group in the population eligible to vote ^5^.

The affective polarization presents wide-ranging implications for our social and economic lives. In concrete terms, partisans with high hostility toward the other party are more motivated to distinguish themselves from their political opponents ^37^. They tend to take positions on new issues that differ from the other (disliked) party and match those of their preferred party. In the context of COVID-19, Druckman et al. ^37^ found a strong association between out-party hostility and subsequent responses to the pandemic, offering evidence that policy beliefs reflect affective feelings toward the other party rather than just the genuine concern of issues at hand.

Despite solid correlations encountered in this study, some limitations must be considered. First, some variables and information used in the model need to be updated (such as the material deprivation index and Pentecostals information provided by the 2010 population census), and more updated information could reduce the statistical association of votes and mortality. However, it would not be so strange to argue that the relative spatial distribution of Pentecostals must not have varied in space over time, and it is possible that their municipal percentage increased (or decreased) in length and over time was constant between places. Regardless, the percentage of Pentecostals in the last 2010 census is a good predictor of excess mortality during the 2020 and 2021 pandemic peaks. Likewise, in addition to the material deprivation index, other socioeconomic controls were not always statistically associated with the peaks of excess mortality.

The second limitation concerns the vote interpretation and the ecological fallacy. The results of this study are based on aggregated data rather than on individuals, which implies that we are not dealing with individual behaviors but rather associations on another unit of analysis. Thus, our main findings represent the average effects of municipal electoral outcomes. However, we highlight that our outcome variable (excess mortality) is only estimated at aggregated levels. In this sense, adhering to this study design does not introduce significant challenges to the identified associations. The third limitation is the lack of updated demographic variables such as age and sex, which are crucial for understanding both mortality patterns and electoral choices.

The fourth and final remark is related to other probable reasons for the loyalty of Bolsonaro voters, which goes beyond the explanatory way of tribal politics and affective polarization. Herein, we can mention the effects of the emergency aid, recent control of fuel prices, etc., which may have aided Bolsonaro’s public support despite his poor political approaches during the pandemic. However, although these limitations exist, this study brings compelling evidence of associations between municipal Brazilian electoral outcomes and demographic events such as excess mortality.

## References

[1] 1. Burki T. COVID-19 in Latin America. Lancet Infect Dis 2020; 20:547-8. 10.1016/S1473-3099(20)30303-0 PMC7164892 32311323

[2] 2. Muñoz N. COVID-19 in Latin America: a first glance to the mortality. Colomb Med (Cali) 2020; 51:e4366. 10.25100/cm.v51i2.4366 PMC7518724 33012894

[3] 3. Rodriguez-Morales AJ, Gallego V, Escalera-Antezana JP, Méndez CA, Zambrano LI, Franco-Paredes C, et al. COVID-19 in Latin America: the implications of the first confirmed case in Brazil. Travel Med Infect Dis 2020; 35:101613. 10.1016/j.tmaid.2020.101613 PMC7129040 32126292

[4] 4. Ajzenman N, Cavalcanti T, Mata D. More than words: leaders' speech and risky behavior during a pandemic. Am Econ J Econ Policy 2023; 15:351-71.

[5] 5. Dreyfuss B, Patir A, Shayo M. On the workings of tribal politics. SSRN 2021; 22 mar. https://papers.ssrn.com/sol3/papers.cfm?abstract_id=3797290.

[6] 6. Viglione G. Four ways Trump has meddled in pandemic science - and why it matters. How US President Donald Trump and his administration have silenced scientists, meddled in their reports, and ignored their advice. Nature News 2020; 3 nov. https://www.nature.com/articles/d41586-020-03035-4. 10.1038/d41586-020-03035-4 33144734

[7] 7. Wallace J, Goldsmith-Pinkham P, Schwartz JL. Excess death rates for Republicans and Democrats during the COVID-19 pandemic. Cambridge: National Bureau of Economic Research; 2022. (NBER Working Paper, 30512).

[8] 8. Stuenkel O. Brazil's polarization and democratic risks. In: Carothers T, Feldmann AE, editors. Divisive politics and democratic dangers in Latin America. Washington DC: Carnegie Endowment for International Peace; 2021. p. 8-12.

[9] 9. Setzler M. Did Brazilians vote for Jair Bolsonaro because they share his most controversial views? Brazilian Political Science Review 2021; 15:e0002.

[10] 10. Guriev S, Papaioannou E. The political economy of populism. J Econ Lit 2020; 60:753-832.

[11] 11. Lima EEC, Gayawan E, Baptista EA, Queiroz BL. Spatial pattern of COVID-19 deaths and infections in small areas of Brazil. PLoS One 2021; 16:e0246808. 10.1371/journal.pone.0246808 PMC7877657 33571268

[12] 12. Lima EEC, Vilela EA, Peralta A, Rocha M, Queiroz BL, Gonzaga MR, et al. Investigating regional excess mortality in selected Latin American countries during the 2020 COVID-19 pandemic. Genus 2021; 77:30. 10.1186/s41118-021-00139-1 PMC8564791 34744175

[13] 13. Castro MC, Kim S, Barberia L, Ribeiro AF, Gurzenda S, Ribeiro KB, et al. Spatiotemporal pattern of COVID-19 spread in Brazil. Science 2021; 372:821-6. 10.1126/science.abh1558 33853971

[14] 14. Xavier DR, Lima E, Silva E, Lara FAE, Silva GRR, Oliveira MF, et al. Involvement of political and socio-economic factors in the spatial and temporal dynamics of COVID-19 outcomes in Brazil: a population-based study. Lancet Reg Health Am 2022; 10:100221. 10.1016/j.lana.2022.100221 PMC8918677 35309089

[15] 15. Hastenreiter Filho HN, Cavalcante LR. Variáveis associadas à mortalidade por covid-19 nos municípios brasileiros: um estudo exploratório. RPER Revista Portuguesa de Estudos Regionais 2022; 60:57-70.

[16] 16. Seara-Morais GJ, Avelino-Silva TJ, Couto M, Avelino-Silva VI. The pervasive association between political ideology and COVID-19 vaccine uptake in Brazil: an ecologic study. BMC Public Health 2023; 23:1606. 10.1186/s12889-023-16409-w PMC10464231 37612648

[17] 17. Backhaus I, Hoven H, Kawachi I. Far-right political ideology and COVID-19 vaccine hesitancy: multilevel analysis of 21 European countries. Soc Sci Med 2023; 335:116227. 10.1016/j.socscimed.2023.116227 37722145

[18] 18. Wallace L, Mikkelborg A, Gonzales R, Hurd K, Romano C, Plaut V. COVID-19 responsibility and blame: how group identity and political ideology inform perceptions of responsibility, blame, and racial disparities. Soc Personal Psychol Compass 2023; 17:e12927.

[19] 19. Panish AR, Ludeke SG, Vitriol JA. Big five personality and COVID-19 beliefs, behaviors, and vaccine intentions: the mediating role of political ideology. Soc Personal Psychol Compass 2024; 18:e12885.

[20] 20. Borah P, Austin E, Su Y. Injecting disinfectants to kill the virus: media literacy, information gathering sources, and the moderating role of political ideology on misperceptions about COVID-19. Mass Commun Soc 2023; 26:566-92.

[21] 21. Castro MC, Gurzenda S, Turra CM, Kim S, Andrasfay T, Goldman N. Reduction in the 2020 life expectancy in Brazil after COVID-19. Nature Med 2021; 27:1629-35. 10.1038/s41591-021-01437-z PMC8446334 34188224

[22] 22. Ministério da Saúde. Mortalidade - desde 1996 pela CID-10. https://datasus.saude.gov.br/mortalidade-desde-1996-pela-cid-10 (accessed on 05/May/2022).

[23] 23. Valerio S. Pentecostalismo, catolicismo e bolsonarismo: convergências. Revista Brasileira de História das Religiões 2020; 13:113-36.

[24] 24. James G, Witten D, Hastie T, Tibshirani R. An introduction to statistical learning: with applications in R. New York: Springer; 2013.

[25] 25. Mathieu E, Ritchie H, Rodés-Guirao L, Appel C, Giattino C, Hasell J, et al. Coronavirus pandemic (COVID-19). https://ourworldindata.org/coronavirus (accessed on 05/May/2022).

[26] 26. Muellbauer J, Aron J. Measuring excess mortality: the case of England during the COVID-19 pandemic. https://www.oxfordmartin.ox.ac.uk/downloads/academic/6-May-20-Muellbauer-Aron-Excess-mortality-in-England-vs.-Europe-and-the-COVID-pandemic.pdf (accessed on 05/May/2022).

[27] 27. Msemburi W, Karlinsky A, Knutson V, Aleshin-Guendel S, Chatterji S, Wakefield J. The WHO estimates of excess mortality associated with the COVID-19 pandemic. Nature 2023; 613:130-7. 10.1038/s41586-022-05522-2 PMC9812776 36517599

[28] 28. Queiroz BL, Lima EEC, Freire FHMA, Gonzaga MR. Temporal and spatial trends of adult mortality in small areas of Brazil, 1980-2010. Genus 2020; 76:36.

[29] 29. Anselin L. Spatial econometrics: methods and models. Amsterdam: Springer Science+Business Media; 1988. (Studies in Operational Regional Science, 4).

[30] 30. Fischer MM, Wang J. Spatial data analysis: models, methods and techniques. New York: Springer; 2011.

[31] 31. Griffith DA. Spatially autoregressive models. In: Kitchin R, Thrift N, editors. International encyclopedia of human geography. Amsterdam: Elsevier; 2009. p. 396-402.

[32] 32. Conselho Nacional de Secretários de Saúde. Painel de análise do excesso de mortalidade por causas naturais no Brasil. https://www.conass.org.br/indicadores-de-obitos-por-causas-naturais/ (accessed on 14/Oct/2022).

[33] 33. Our World in Data. Coronavirus pandemic (COVID-19). https://ourworldindata.org/coronavirus (accessed on 10/Oct/2022).

[34] 34. Anselin L, Gallo J, Jayet H. Spatial panel econometrics. In: Mátyás L, Sevestre P, editors. The econometrics of panel data. Berlin: Springer; 2008. p. 62-60.

[35] 35. Franco AB, Pound N. The foundations of Bolsonaro's support: exploring the psychological underpinnings of political polarization in Brazil. J Community Appl Soc Psychol 2022; 32:846-59.

[36] 36. Maia AG, Marteleto L, Rodrigues CG, Sereno LG. The short-term impacts of coronavirus quarantine in São Paulo: the health-economy trade-offs. PLoS One 2021; 16:e0245011. 10.1371/journal.pone.0245011 PMC7888633 33596219

[37] 37. Druckman JN, Klar S, Krupnikov Y, Levendusky M, Ryan JB. Affective polarization, local contexts and public opinion in America. Nat Hum Behav 2021; 5:28-38. 10.1038/s41562-020-01012-5 33230283

[38] 38. Departamento de Informática do SUS. Mortalidade - desde 1996 pela CID-10. http://tabnet.datasus.gov.br/cgi/deftohtm.exe?sim/cnv/obt10br.def (accessed on 05/Jul/2022).

[39] 39. Ipeadata. Regional - eleições para presidente 2018. http://www.ipeadata.gov.br/Default.aspx (accessed on 05/May/2021).

[40] 40. Ipeadata. Regional - eleições para presidente 2022. http://www.ipeadata.gov.br/Default.aspx (accessed on 01/Mar/2023).

[41] 41. Ministério da Previdência Social. Quantidade de benefícios emitidos pelo Instituto Nacional do Seguro Social - INSS, nos municípios brasileiros, segundo grupos de espécies Dezembro 2021. https://www.gov.br/previdencia/pt-br/assuntos/previdencia-social/emitidos-municipios-2021 (accessed on 05/May/2022).

[42] 42. Instituto Brasileiro de Geografia e Estatística. Produto interno bruto dos municípios em 2019. https://www.ibge.gov.br/estatisticas/economicas/contas-nacionais/9088-produto-interno-bruto-dos-municipios.html?edicao=32575&t=resultados (accessed on 03/May/2022).

[43] 43. Secretaria de Avaliação, Gestão da Informação e Cadastro Único. Famílias inscritas no Cadastro Único - quantidade total e por faixa de renda familiar per capita em 2020. https://aplicacoes.cidadania.gov.br/vis/data3/data-explorer.php# (accessed on 03/May/2022).

[44] 44. Secretaria de Avaliação, Gestão da Informação e Cadastro Único. Famílias inscritas no Cadastro Único - quantidade total e por faixa de renda familiar per capita em 2021. https://aplicacoes.cidadania.gov.br/vis/data3/data-explorer.php# (accessed on 03/May/2022).

[45] 45. Ministério do Trabalho e Emprego. Cadastro Geral de Empregados e Desempregados - CAGED. https://portalfat.mte.gov.br/cadastro-geral-de-empregados-e-desempregados-caged/ (accessed on 10/Sep/2021).

[46] 46. Instituto Brasileiro de Geografia e Estatística. Microdados do Censo Demográfico 2010. https://www.ibge.gov.br/estatisticas/sociais/populacao/9662-censo-demografico-2010.html?=&t=microdados (accessed on 03/May/2022).

[47] 47. Departamento de Informática do SUS. CNES - Estabelecimentos - Classificação do serviço - Brasil em 2020. http://tabnet.datasus.gov.br/cgi/tabcgi.exe?cnes/cnv/servc2br.def (accessed on 14/Sep/2022).

[48] 48. Departamento de Informática do SUS. CNES - Estabelecimentos - Classificação do serviço - Brasil em 2021. http://tabnet.datasus.gov.br/cgi/tabcgi.exe?cnes/cnv/servc2br.def (accessed on 14/Sep/2022).

[49] 49. Departamento de Informática do SUS. CNES - Estabelecimentos por nível de atenção - Brasil. Ambulatorial - Básica municipal, segundo município em 2020. http://tabnet.datasus.gov.br/cgi/tabcgi.exe?cnes/cnv/atencbr.def (accessed on 14/Sep/2022).

[50] 50. Departamento de Informática do SUS. CNES - Estabelecimentos por nível de atenção - Brasil. Ambulatorial - Básica municipal, segundo município em 2021. http://tabnet.datasus.gov.br/cgi/tabcgi.exe?cnes/cnv/atencbr.def (accessed on 14/Sep/2022).

[51] 51. Departamento de Informática do SUS. Dados detalhados das AIH - Por Residência - Brasil. Quantidade aprovada segundo município em 2020. http://tabnet.datasus.gov.br/cgi/tabcgi.exe?sih/cnv/sprbr.def (accessed on 14/Sep/2022).

[52] 52. Departamento de Informática do SUS. Dados detalhados das AIH - Por residência - Brasil. Quantidade aprovada segundo município em 2021. http://tabnet.datasus.gov.br/cgi/tabcgi.exe?sih/cnv/sprbr.def (accessed on 14/Sep/2022).

[53] 53. Departamento de Informática do SUS. CNES - Recursos físicos - hospitalar - leitos de internação - Brasil. Quantidade existente por município em 2020. http://tabnet.datasus.gov.br/cgi/deftohtm.exe?cnes/cnv/leiintbr.def (accessed on 14/Sep/2022).

[54] 54. Departamento de Informática do SUS. CNES - Recursos físicos - hospitalar - Leitos de internação - Brasil. Quantidade existente por município em 2021. http://tabnet.datasus.gov.br/cgi/deftohtm.exe?cnes/cnv/leiintbr.def (accessed on 14/Sep/2022).

[55] 55. Departamento de Informática do SUS. Cobertura - Brasil. Coberturas vacinais segundo município em 2020. http://tabnet.datasus.gov.br/cgi/dhdat.exe?bd_pni/cpnibr.def (accessed on 14/Sep/2022).

