# Supplementary material for: Presidential election results in 2018-2022 and its association with excess mortality during the 2020-2021 COVID-19 pandemic in Brazilian municipalities
Source: Cad Saude Publica. 2024 Jun 14;40(5):e00194723. doi: 10.1590/0102-311XEN194723 (PMC11178372; doi:10.1590/0102-311XEN194723)
Supplement: Supplementary file 1 [file 1678-4464-csp-40-05-EN194723-s.pdf]

Table S1 Lagrange multiplier test diagnostics for spatial dependence and regression models to explain municipal excess mortality in Brazil during the 2020-2021 COVID-19 pandemic. Workers' Party'S (PT) first-round votes 2018-2022.

Lagrange multiplier test diagnostics for spatial dependence, PT's first-round votes 2018-2022

Type of spatial dependency test	2018 votes and 1st peak mortality		2022 votes and 1st peak mortality		2018 votes and 2nd peak mortality		2022 votes and 2nd peak mortality	
	Statistic	p-value	Statistic	p-value	Statistic	p-value	Statistic	p-value
Lagrange multiplier error model	24.91	0.000	24.06	0.000	25.51	0.000	26.06	0.000
Lagrange multiplier lag model	22.03	0.000	21.15	0.000	19.18	0.000	19.55	0.000
Robust Lagrange multiplier error model	3.41	0.065	3.51	0.061	7.18	0.007	7.42	0.006
Robust Lagrange multiplier lag model	0.53	0.465	0.60	0.440	0.85	0.357	0.91	0.340

Regression models to explain municipal excess mortality in Brazil in the function of first-round votes for PT candidate

Control variables	2018 PT votes – 1st peak excess deaths				2022 PT votes – 1st peak excess deaths			
	Linear model		Spatial error model		Linear model		Spatial error model	
	β	p-value	β	p-value	β	p-value	β	p-value
Intercept	43.69	0.001	44.75	0.001	71.59	0.000	72.35	0.000
% first-round votes	-0.18	0.074	-0.20	0.063	-0.38	0.001	-0.39	0.001
Material deprivation index	-1.72	0.537	-1.09	0.709	-0.33	0.898	0.06	0.981
GDP	0.08	0.610	0.07	0.661	0.09	0.574	0.08	0.630
Extreme poverty ratio	-3.19	0.350	-2.96	0.400	-3.78	0.269	-3.45	0.326
% age retirement	0.09	0.314	0.08	0.404	0.07	0.409	0.06	0.513
% Pentecostals affiliated	0.70	0.000	0.71	0.000	0.62	0.000	0.63	0.000
Health establishments	-0.004	0.391	-0.003	0.463	-0.004	0.378	-0.003	0.446
Health outpatient clinics	0.01	0.657	0.01	0.670	0.01	0.656	0.01	0.668
Longitude	-0.14	0.484	-0.12	0.576	0.07	0.730	0.10	0.676
Latitude	1.18	0.000	1.14	0.000	1.26	0.000	1.23	0.000
	R^2 = 0.015		Lambda: 0.019 *		R^2 = 0.017		Lambda: 0.018 *	
	AIC: 63,194		AIC: 63,173		AIC: 63,209		AIC: 63,188	

Control variables	2018 PT votes – 2nd peak excess deaths				2022 PT votes – 2nd peak excess deaths			
	Linear model		Spatial error model		Linear model		Spatial error model	
	β	p-value	β	p-value	β	p-value	β	p-value
Intercept	11,88	0.545	11.35	0.588	33.87	0.171	33.49	0.203
% first-round votes	-0.50	0.001	-0.50	0.003	-0.47	0.006	-0.48	0.009
Material deprivation index	-8.93	0.036	-8.11	0.069	-11.81	0.003	-10.79	0.009
GDP	0.19	0.459	0.22	0.409	0.20	0.433	0.22	0.395
Extreme poverty ratio	9.77	0.065	8.81	0.106	9.73	0.067	8.79	0.107
% age retirement	0.04	0.767	0.04	0.786	0.01	0.967	0.00	0.975
% Pentecostals affiliated	1.12	0.000	1.04	0.000	1.07	0.000	1.00	0.000
Health facilities	-0.004	0.545	-0.005	0.463	0.00	0.522	-0.005	0.449
Health outpatient clinics	-0.01	0.679	-0.01	0.717	-0.01	0.686	-0.01	0.722
Longitude	-0.99	0.001	-1.08	0.001	-0.77	0.019	-0.85	0.017
Latitude	0.48	0.148	0.45	0.209	0.63	0.058	0.59	0.102
	R^2 = 0.037		Lambda: 0.019 *		R^2 = 0.036		Lambda: 0.019 *	
	AIC: 67,951		AIC: 67,928		AIC: 67,979		AIC: 67,956	

AIC: Akaike information criterion; GDP: gross domestic product.

Sources: Brazilian Ministry of Health (2022), Brazilian Superior Electoral Court (2018 and 2022), Brazilian Ministry of Labor and Social Security (2021), Brazilian Ministry of Citizenship (2019), Brazilian Institute of Geography and Statistics (2010 and 2019).

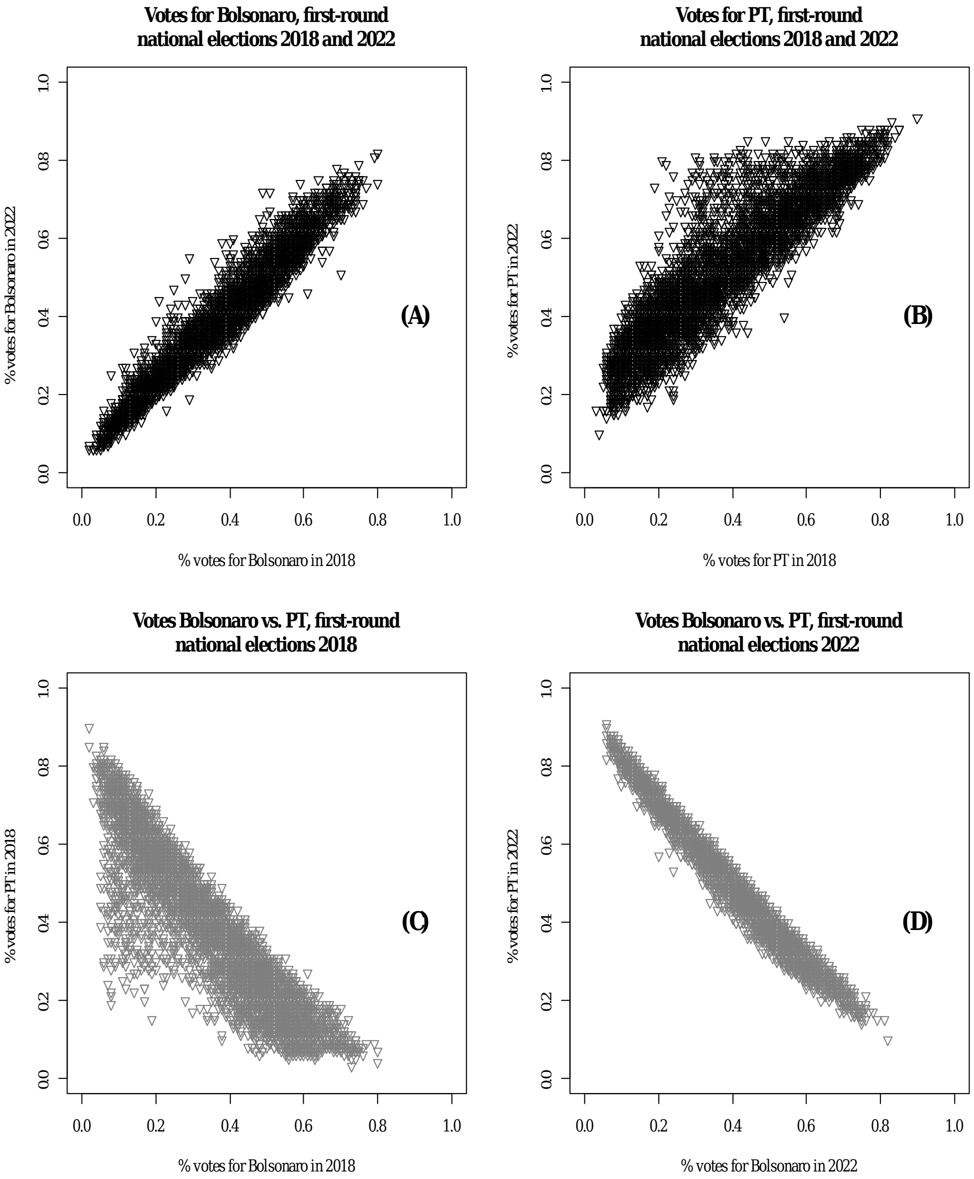

Figure S1 Relationship acroos municipalities between votes for Bolsonaro and Worker's Party (PT), first round Brazilian elections 2018 and 2022.

Source: Superior Electoral Court, 2018 and 2022.
